# The Effects of Greek Orthodox Christian Fasting during Holy Week on Body Composition and Cardiometabolic Parameters in Overweight Adults

**DOI:** 10.3390/diseases10040120

**Published:** 2022-12-05

**Authors:** Kalliopi Georgakouli, Fotini Siamata, Dimitrios Draganidis, Panagiotis Tsimeas, Konstantinos Papanikolaou, Alexios Batrakoulis, Athanasios Gatsas, Athanasios Poulios, Niki Syrou, Chariklia K. Deli, Ioannis G. Fatouros, Athanasios Z. Jamurtas

**Affiliations:** 1 Department of Nutrition and Dietetics, University of Thessaly, Argonauton 1C, 42132 Trikala, Greece; 2 Department of Physical Education and Sport Science, University of Thessaly, Karies, 42100 Trikala, Greece

**Keywords:** religion, diet, body composition, metabolism, heart, health

## Abstract

This study investigated whether Greek Orthodox Christian fasting during Holy Week can change body composition and cardiometabolic parameters in overweight individuals, and whether these changes are maintained one week after fasting cessation (FC). Body composition and physiological and biochemical parameters were measured before, immediately after (n = 23) and one week after FC (subgroup of n = 10). Fasting resulted in decreased body weight, waist circumference, waist-to-hip ratio, body mass index and total body fat, as well as blood glucose, total cholesterol and low-density lipoprotein cholesterol levels. Nutrition analysis showed a decreased protein and saturated fat intake during fasting. FC (n = 10) resulted in a decreased carbohydrate intake and increased protein and cholesterol intake compared to fasting. Fasting resulted in decreased blood glucose, total cholesterol and LDL cholesterol levels but returned to pre-fasting levels after FC. Greek Orthodox Christian fasting during Holy Week is beneficial for body composition and some aspects of cardiometabolic health. However, these favourable changes are not maintained one week following fasting.

## 1. Introduction

Cardiometabolic diseases includes cardiovascular disease and diabetes mellitus (DM) and are a leading cause of death around the world [1]. Lifestyle factors, such as diet, physical activity and sleep, influence changes in cardiometabolic parameters and the risk of the development of cardiometabolic disease. Poor dietary habits have a negative influence on cardiometabolic parameters [2], with various dietary factors shown to be associated with mortality from heart disease, stroke and type 2 DM [3].

Vegetarian diets are associated with decreased concentrations of total and LDL cholesterol and increased concentrations of HDL cholesterol, but do not affect triglyceride concentrations [4,5]. Moreover, vegetarian diets are associated with reduced body weight and improved obesity-related inflammatory profiles suggesting that they contribute to the prevention and management of weight-related health problems [6,7,8], including type 2 DM [9].

Fasting is the abstinence from certain or all foods during specific days or periods of time, a common practice in many religions. The diet restrictions for Greek Orthodox Christians depend on the day of the week and the period of the year. Greek Orthodox Christians are generally advised to refrain from meat, fish, eggs, milk, dairy products and olive oil every Wednesday and Friday, while they are advised to fast most of the days during Nativity (40 days before Christmas) and Lent (48 days preceding Easter). Additionally, 15 days preceding the Assumption of Mary, they follow a vegetarian diet pattern that excludes meat, fish (except for shellfish), eggs and dairy products [10]. Studies on the effects of Greek Orthodox Christian fasting are scarce. The available data indicate that Greek Orthodox Christian fasting results in reduced body weight and some favourable changes in serum lipids (decreased total and LDL cholesterol) in regular fasters [10] and increased antioxidant status (total antioxidant capacity) following a 48-day fast before Easter [11]. Moreover, a previous study by Papadaki and colleagues in 2008 [12] showed that blood lipid levels were more favourable during a fasting week (Palm Sunday) than during a week in which a normal diet was followed (the week following Pentecost Sunday) in Greek Orthodox Christian monks [12]. However, no study has examined how long the favourable changes observed in the aforementioned studies are maintained after fasting cessation.

The purpose of this study was to examine the following: (1) the effects of short-term Greek Orthodox Christian fasting (during Holy Week) on body composition characteristics and cardiometabolic parameters (blood glucose and lipids, blood pressure); and (2) whether these effects are still evident one week after fasting cessation.

## 2. Materials and Methods

### 2.1. Subjects

Twenty-three (4 men, 19 women) overweight [body mass index (BMI) ≥ 25 kg/m^2^] adults volunteered to participate in this study and provided informed consent. A questionnaire regarding medical history, medication and family history was completed. Participation criteria included an absence of metabolic diseases, pregnancy and medication over the last 6 months. The baseline characteristics of the participants are presented in Table 1. The procedures were conducted in accordance with the 1975 Declaration of Helsinki and its later amendments, and the study protocol was approved by the Ethics Committee of the Department of Physical Education and Sport Science, University of Thessaly (Protocol Number: 1163).

### 2.2. Experimental Design

All participants (n = 23) reported to the laboratory twice, before (pre-fasting state; pre-F) and immediately after Holy Week (post-fasting state; post-F). Moreover, 10 of the participants reported to the laboratory once more, one week after fasting cessation (non-fasting state, NF). Body composition, physiological measurements and blood samples were obtained at pre-F, post-F and NF. Blood was later analysed for glucose, uric acid, total cholesterol, low-density lipoprotein (LDL) cholesterol, high-density lipoprotein (HDL) cholesterol and triglycerides in serum. For three days before (pre-F) and three days during Holy Week (fasting; F), as well as for three days after one week of fasting cessation (NF), participants recorded their diet. The diet followed by the participants shared some similarities with the Mediterranean diet in terms of the increased consumption of olive oil and plant foods (starchy foods, legumes, cereals, nuts, vegetables, fruits). However, milk, dairy products (including butter), eggs, meat and fish were excluded, while seafood, except for fish, was allowed (i.e., molluscs and crustaceans). Dietary records were later analysed using ScienceFit Diet 200A (Science Technologies, Athens, Greece).

### 2.3. Blood Collection and Handling

Blood samples (10 mL each) were drawn from a forearm vein following an overnight fast. Each blood sample was transferred into a separate tube containing a clot activator, left at room temperature for 20 min to clot, and then centrifuged at 1370× *g* for 10 min at 4 °C in order to obtain the supernatant (serum) that was aliquoted into Eppendorf™ tubes. Serum was stored at −80 °C for the later determination of glucose, uric acid, total cholesterol, HDL cholesterol and triglycerides. Each sample underwent only one freeze–thaw cycle, and each parameter was analysed in duplicates on the same day.

### 2.4. Biochemical Assays

Glucose was measured by the GOD/POD/PAP enzymatic colorimetric method, total cholesterol was measured by the CHOD-PAP enzymatic colorimetric method, HDL cholesterol was measured by the method of precipitation of phospho-fumaric acid-MgCl_2_ and triglycerides were measured by the GPO-PAP enzymatic colorimetric method in a biochemical analyser (CLINICAL CHEMISTRY ANALYZER Z 1145-ZAFIROPOULOS DIAGNOSTICA) with reagents from P. Zafiropoulos S.A. (Athens, Greece). LDL cholesterol was estimated using the Friedewald equation [13].

### 2.5. Body Composition Measurements

The participants were lightly dressed and barefoot, standing height was measured to the nearest 0.5 cm (Stadiometer 208; Seca, Birmingham, UK) and body mass was measured to the nearest 0.1 kg (Beam Balance 710; Seca, Birmingham, UK). BMI was calculated using the following equation: BMI = body weight/height^2^. Body fat percentage (%fat) was measured with a Tanita Body Fat Monitor/Scale TBF-521 (Tanita, Inc., Arlington Heights, IL, USA). Waist and hip circumferences were obtained with a measuring tape. Waist-to-hip ratio (WHR) was calculated using the following equation: WHR = waist circumference/hip circumference.

### 2.6. Physiological Measurements

Blood pressure (BP) was measured with participants in a sitting position using a manual sphygmomanometer (FC-101 Aneroid Sphygmomanometer; Focal Corporation, Kashiwa, Japan) after 5 min of rest.

### 2.7. Statistical Methods

Statistical analysis was performed with IBM SPSS Version 19.0 (IBM Corp., Armonk, NY, USA). A Shapiro–Wilk test was used to check the normality of the variables. Because not all variables were normally distributed, we applied non-parametric tests. A Wilcoxon signed-rank test was performed to compare pre-F and post-F values in all 23 participants. The Friedman analysis of variance by ranks test was performed to compare pre-F, post-F (or during F for dietary analysis) and one-week post-fasting (NF) values in 10 participants, accompanied by the Wilcoxon signed-rank test to perform pairwise comparisons. The level of statistical significance was set at *p* < 0.05. Data are presented as means ± SD.

## 3. Results

### 3.1. Body Composition and Physiological Characteristics

Table 1 shows the differences in the anthropometric characteristics of the participants at pre-F and post-F. There were significant decreases in weight (−0.6%, z = −2.94, *p* = 0.003), BMI (−0.7%, z = −3.05, *p* = 0.002) and %fat (−2.5%, z = −2.36, *p* = 0.018). Moreover, there was a non-significant decrease in waist circumference (−0.7%, z = −1.94, *p* = 0.053) and WHR (−0.4%, z = −1.90, *p* = 0.057).

Table 2 shows the results from the comparison of the pre-F, post-F and NF values of the anthropometric characteristics. A significant increase (4.2%, z = −2.71, *p* = 0.07) in %fat at NF compared to the immediately post-F values was found.

### 3.2. Biochemical Parameters

Comparison of the pre-F and post-F values of the biochemical parameters are shown in Table 3. There was a significant decrease in glucose (−5.7%, z = −3.15, *p* = 0.002), total cholesterol (−8.1%, z = −3.27, *p* = 0.001) and LDL cholesterol (−13.7%, z = −2.37, *p* = 0.018), whereas triglycerides and HDL cholesterol did not change.

Table 4 shows the results from the comparison of the pre-F, post-F and NF values of the biochemical parameters. Regarding glucose, there was a significant decrease (−6.1%, z = −2.29, *p* = 0.022) at post-F compared to the pre-F values, and then a significant (7.3%, z = −2.80, *p* = 0.005) increase at NF compared to the post-F values. Regarding LDL cholesterol, there was a significant decrease (−14.5%, z = −2.45, *p* = 0.014) at post-F compared to the pre-F values, and then a significant (29.2%, z = −2.81, *p* = 0.005) increase at NF compared to the post-F values. Total cholesterol decreased significantly (−10.0%, z = −2.45, *p* = 0.014) at post-F compared to the pre-F values, and then increased significantly (19.3%, z = −2.81, *p* = 0.005) at NF compared to the post-F values.

### 3.3. Diet Analysis

Comparison of the pre-F and F values of daily energy and macronutrient intake are shown in Table 5. Protein (−28.7%, z = −3.02, *p* = 0.003) and saturated fat intake (−18.5%, z = −2.03, *p* = 0.042) decreased during F.

Table 6 shows the results from the one-way repeated measures ANOVA for the pre-F, F and NF values of energy and macronutrients. Carbohydrates (g) decreased significantly at NF compared to pre-F (−22.2%, z = −2.19, *p* = 0.028) and F (−27.3%, z = −1.99, *p* = 0.047). Proteins (g) decreased significantly at F compared to pre-F (−37.2%, z = −2.31, *p* = 0.021) and increased significantly at NF compared to F (52.1%, z = −2.19, *p* = 0.028). Cholesterol (mg) decreased significantly during F compared to pre-F (−51.4%, z = −2.07, *p* = 0.038) and increased significantly at NF compared to both pre-F (54.1%, z = −2.67, *p* = 0.008) and F (216.9%, z = −2.80, *p* = 0.005).

## 4. Discussion

In this study, we investigated the effects of Greek Orthodox Christian fasting during Holy Week on body composition changes and cardiometabolic parameters (blood glucose, lipids and blood pressure) in overweight individuals and examined whether these effects were maintained one week after fasting cessation. The primary findings of this study suggest that Greek Orthodox Christian fasting during Holy Week is beneficial for body composition changes and some aspects of cardiometabolic health. However, these favourable changes are not maintained a week following fasting cessation.

An abnormal blood lipid profile is an important cardiometabolic risk factor. In the present study, one week of Greek Orthodox Christian fasting resulted in decreased total and LDL cholesterol. Previous studies on Greek Orthodox Christian fasting have also shown some favourable changes in blood lipids (decreased total and LDL cholesterol, no change in triglycerides and HDL cholesterol) [10]. This could be attributed to low saturated fat intake, which is associated with a low risk of cardiovascular disease [14]. In the present study, a 18.5% decrease in saturated fat intake was found during fasting compared to pre-fasting, which is in accordance with previous reports regarding Greek Orthodox Christian fasting [12,15].

Moreover, total and LDL cholesterol decreased following one week of fasting and returned to baseline levels one week after fasting cessation. Similarly, it has been previously observed that blood lipid levels are more favourable during the fasting week [12]. Therefore, one can assume that the more days of the year one follows Greek Orthodox Christian fasting, the more benefits in the blood lipid profile will be gained.

Diet is an important component of normal glucose metabolism [16]. Decreased carbohydrate consumption may reduce insulin release and resistance, resulting in improved glycaemic control. However, evidence from prospective observational studies suggests that the relative carbohydrate proportion of a diet may not influence diabetes risk [17]. On the other hand, a diet rich in fibre, especially cereal fibre, may reduce diabetes risk [18]. In the present study, blood glucose levels decreased following Greek Orthodox Christian fasting during the Holy Week but returned to baseline levels one week after fasting cessation, whereas triglycerides remained unchanged. Although changes in triglyceride levels over time could be used as a predictor of type 2 DM [19], one week of diet manipulation in non-diabetic individuals may not be enough to cause such changes. Previous studies on the effect of Greek Orthodox Christian fasting on blood glucose and triglyceride levels have provided conflicting results [10]. Moreover, carbohydrate consumption did not change, but fibre consumption increased (by 5 g/day) during Greek Orthodox Christian fasting, as observed before [12,20]. Taken together, it is possible that fibre intake is an important dietary factor that contributes to decreased cardiometabolic disease risk. The glycaemic index and load of the diet could also have played a role.

Participants were overweight according to BMI. Greek Orthodox Christian fasting during the Holy Week resulted in a small yet significant decrease in body mass and BMI, but one week following fasting cessation, these parameters returned to baseline values. This is in accordance with a previous study [20] that reported a 10% reduction in energy intake along with a reduction in BMI during fasting, but these effects were not sustained in non-fasting periods. The association between BMI and the risk of cardiometabolic disease is well documented [21]. Thus, theoretically, this reduction in BMI during fasting could have a positive impact on cardiometabolic health; however, in the present study, no significant reduction in daily energy intake during fasting was found. It is possible that BMI reduction was the result of increased energy expenditure rather than decreased energy intake.

De novo lipogenesis is a biosynthetic pathway in the liver that contributes to fat storage and secretion by hepatocytes [22]. De novo lipogenesis converts excess carbohydrates (from carbohydrate metabolism or diet) into fatty acids and then to triglycerides; therefore, the rate of this process is higher when a high-carbohydrate diet is consumed [23]. Various factors contribute to fat accumulation in the liver, including increased fat availability from visceral fat tissue [24] and increased de novo lipogeneses due to peripheral insulin resistance [25]. On the other hand, the Mediterranean diet has been shown to regulate these processes [26] due to the antioxidant, anti-inflammatory and antifibrotic effects of polyphenols and monounsaturated fatty acids that are consumed in large amounts in this diet [27]. In the present study, dietary analysis showed that the synthesis of Greek Orthodox Christian fasting was similar to that of the Mediterranean diet in terms of macronutrient ratio (moderate carbohydrate, low protein and high fat intake, with particularly high monosaturated fat intake). Long-term adherence to such a dietary pattern may be affective in lowering liver fat accumulation, which is associated with an increased risk of various health problems, including non-alcoholic fatty liver disease [27].

Increased blood pressure contributes to cardiovascular disease. Various dietary factors affect blood pressure. Dietary changes that decrease blood pressure include weight loss, increased potassium intake and vegetarian dietary patterns [28]. In the present study, participants were normotensive and no change in blood pressure was observed following Greek Orthodox Christian fasting. Research on the effects of Greek Orthodox Christian fasting on blood pressure is scarce and the results are conflicting [12,29]. One previous study found that during fasting, systolic blood pressure increased and diastolic blood pressure did not change [12], whereas another study found that fasters had a higher mean systolic and diastolic blood pressure than non-fasters and that fasting did not cause any change [29]. Studies in hypertensive individuals are warranted to identify possible changes in blood pressure due to Greek Orthodox Christian fasting.

Greek Orthodox Christian fasting is a diet pattern of dietary restrictions, involving a decrease in one or more components of dietary intake with minimal to no decrease in total caloric intake [10]. Dietary changes observed during fasting in fibre consumption and saturated fatty acids can explain the beneficial effects of Greek Orthodox Christian fasting on cardiometabolic risk factors, such as BMI, blood glucose, total cholesterol and LDL cholesterol. In addition, decreased protein consumption observed following fasting might also result in beneficial health effects [30]. Finally, because fasting is a plant-based diet, it is possible that dietary factors found in vegetables and fruits, including antioxidants and phytochemicals, may also have a positive impact on health [31,32]. Nevertheless, it must be mentioned that the cardiometabolic benefits of fasting dissipated after only a week of fasting cessation. Long-term adherence to plant-based diets should be promoted as they have shown to improve cardiometabolic parameters, such as body weight and endothelial factors [6,33].

## 5. Conclusions

During Greek Orthodox Christian fasting, a vegetarian diet pattern is followed. Changes in the consumption of various dietary factors can favourably affect risk factors of cardiometabolic health. These positive effects attenuate after fasting cessation and sustained dietary changes would provide more beneficial effects. More research on the long-term effects of periodical fasting on health parameters and nutritional status is warranted.

## Figures and Tables

**Table 1:** Differences in body composition and physiological characteristics at pre-F and post-F (n = 23).

Characteristic	Pre-F	Post-F
Weight (kg)	74.2 ± 14.7	73.7 ± 14.8 *
Waist circumference (cm)	85.7 ± 11.7	85.0 ± 11.3
Hip circumference (cm)	105.5 ± 8.6	105.3 ± 8.3
WHR	0.811 ± 0.08	0.807 ± 0.08
BMI (kg/m^2^)	27.6 ± 5.4	27.4 ± 5.4 *
Body fat (%)	32.0 ± 0.08	31.0 ± 0.08 *
Systolic BP (mm Hg)	109.7 ± 13.4	109.0 ± 12.8
Diastolic BP (mm Hg)	69.3 ± 8.6	67.0 ± 8.7

* Significant difference (*p* < 0.05) from immediately post-F. WHR: waist-to-hip ratio; BMI: body mass index; BP: blood pressure.

**Table 2:** Differences in body composition and physiological characteristics at pre-F, post-F and NF (n = 10).

Characteristic	Pre-F	Post-F	NF
Weight (kg)	71.4 ± 9.0	71.1 ± 9.4	71.2 ± 9.0
Waist circumference (cm)	83.4 ± 8.9	83.0 ± 8.9	82.3 ± 9.0
Hip circumference (cm)	101.7 ± 5.9	101.6 ± 5.6	101.5 ± 6.2
WHR	0.82 ± 0.09	0.82 ± 0.09	0.81 ± 0.10
BMI (kg/m^2^)	25.7 ± 3.3	25.6 ± 3.4	25.6 ± 3.3
Body fat (%)	28.7 ± 7.0	27.8 ± 6.2	29.0 ± 6.7 *
Systolic BP (mm Hg)	113.3 ± 14.9	113.3 ± 12.9	109.4 ± 10.5
Diastolic BP (mm Hg)	71.8 ± 9.8	71.5 ± 9.1	71.3 ± 7.7

* Significant difference (*p* < 0.05) from post-F. WHR: waist-to-hip ratio; BMI: body mass index; BP: blood pressure.

**Table 3:** Differences in biochemical parameters pre-F and post-F (n = 23).

Parameter	Pre-F	Post-F
Glucose (mg/dL)	79.9 ± 7.2	75.3 ± 7.7 *
Triglycerides (mg/dL)	79.1 ± 15.4	80.0 ± 23.4
Total cholesterol (mg/dL)	160.5 ± 24.1	147.5 ± 22.5 *
LDL cholesterol (mg/dL)	89.6 ± 25.5	77.4 ± 22.3 *
HDL cholesterol (mg/dL)	55.0 ± 10.5	54.7 ± 9.7

* Significant difference (*p* < 0.05) from post-F. WHR: waist-to-hip Ratio; BMI: body mass index; BP: blood pressure.

**Table 4:** Differences in biochemical parameters at pre-F, post-F and NF (n = 10).

Parameter	Pre-F	Post-F	NF
Glucose (mg/dL)	80.9 ± 9.1	75.9 ± 9.5 ^#^	81.4 ± 8.7 *
Triglycerides (mg/dL)	76.1 ± 17.8	73.1 ± 24.2	73.1 ± 24.2
Total cholesterol (mg/dL)	163.0 ± 24.4	146.8 ± 20.5 ^#^	175.1 ± 26.4 *
LDL cholesterol (mg/dL)	91.8 ± 24.7	78.5 ± 21.2 ^#^	101.4 ± 29.3 *
HDL cholesterol (mg/dL)	56.0 ± 8.3	53.7 ± 10.7	58.0 ± 13.0

* Significant difference (*p* < 0.05) from post-F. ^#^ Significant difference (*p* < 0.05) from pre-F. WHR: waist-to-hip Ratio; BMI: body mass index; BP: blood pressure.

**Table 5:** Differences in daily energy and macronutrient intake at pre-F and F (n = 23).

Variable	Pre-F	F
Energy (kcal)	1964.0 ± 592.8	2059.9 ± 752.0
Carbohydrates (%)	45.9	50.0
Carbohydrates (g)	225.5 ± 82.5	257.3 ± 89.4
Proteins (%)	14.1	9.6
Proteins (g)	69.3 ± 20.1	49.4 ± 17.6 *
Fats (%)	38.9	41.1
Fats (g)	84.9 ± 29.3	94.1 ± 48.6
-saturated (g)	20.0 ± 6.5	16.3 ± 7.1 *
-monounsaturated (g)	45.6 ± 20.0	57.2 ± 35.0
-polyunsaturated (g)	10.9 ± 3.8	14.0 ± 6.1
Cholesterol (mg)	186.5 ± 81.5	155.0 ± 128.2
Fibre (g)	22.4 ± 9.5	25.6 ± 9.0

* Significant difference (*p* < 0.05) from pre-F. WHR: waist-to-hip ratio.

**Table 6:** Differences in daily energy and macronutrient intake at pre-F, F and NF (n = 10).

Variable	Pre-F	F	NF
Energy (kcal)	2198.0 ± 657.7	2379.4 ± 914.6	2151.2 ± 472.8
Carbohydrates (%)	46.0	45.5	36.6
Carbohydrates (g)	252.9 ± 98.9	270.8 ± 103.7	196.8 ± 35.9 *^#^
Proteins (%)	15.0	8.7	14.7
Proteins (g)	82.5 ± 17.6	51.8 ± 15.4 ^#^	78.8 ± 17.4 *
Fats (%)	39.9	48.5	48.5
Fats (g)	97.5 ± 36.3	128.2 ± 56.9	116.0 ± 40.0
-saturated (g)	22.2 ± 6.4	19.7 ± 7.9	26.6 ± 10.2
-monounsaturated (g)	56.2 ± 26.9	84.3 ± 40.8	65.5 ± 26.2
-polyunsaturated (g)	12.7 ± 4.4	15.8 ± 6.4	13.5 ± 3.7
Cholesterol (mg)	218.1 ± 85.8	106.1 ± 100.9#	336.2 ± 42.5 *^#^
Fibre (g)	28.3 ± 10.2	35.0 ± 17.9	23.6 ± 4.7

* Significant difference (*p* < 0.05) from F. ^#^ Significant difference (*p* < 0.05) from pre-F. WHR: waist-to-hip Ratio; BMI: body mass index; BP: blood pressure.

## Data Availability

Not applicable.
